# Relationships of Glucose, GLP-1, and Insulin Secretion With Gastric Emptying After a 75-g Glucose Load in Type 2 Diabetes

**DOI:** 10.1210/clinem/dgac330

**Published:** 2022-05-24

**Authors:** Ryan J Jalleh, Tongzhi Wu, Karen L Jones, Christopher K Rayner, Michael Horowitz, Chinmay S Marathe

**Affiliations:** Endocrine and Metabolic Unit, Royal Adelaide Hospital, Adelaide, Australia; Adelaide Medical School, The University of Adelaide, Adelaide, Australia; Centre of Research Excellence in Translating Nutritional Science to Good Health, The University of Adelaide, Adelaide, Australia; Adelaide Medical School, The University of Adelaide, Adelaide, Australia; Centre of Research Excellence in Translating Nutritional Science to Good Health, The University of Adelaide, Adelaide, Australia; Endocrine and Metabolic Unit, Royal Adelaide Hospital, Adelaide, Australia; Adelaide Medical School, The University of Adelaide, Adelaide, Australia; Centre of Research Excellence in Translating Nutritional Science to Good Health, The University of Adelaide, Adelaide, Australia; Adelaide Medical School, The University of Adelaide, Adelaide, Australia; Centre of Research Excellence in Translating Nutritional Science to Good Health, The University of Adelaide, Adelaide, Australia; Endocrine and Metabolic Unit, Royal Adelaide Hospital, Adelaide, Australia; Adelaide Medical School, The University of Adelaide, Adelaide, Australia; Centre of Research Excellence in Translating Nutritional Science to Good Health, The University of Adelaide, Adelaide, Australia; Endocrine and Metabolic Unit, Royal Adelaide Hospital, Adelaide, Australia; Adelaide Medical School, The University of Adelaide, Adelaide, Australia; Centre of Research Excellence in Translating Nutritional Science to Good Health, The University of Adelaide, Adelaide, Australia

**Keywords:** insulin secretion, gastric emptying, type 2 diabetes, glucose tolerance test, glycemia, C-peptide

## Abstract

**Context:**

The relationships of gastric emptying (GE) with the glycemic response at 120 minutes, glucagon-like peptide-1 (GLP-1), and insulin secretion following a glucose load in type 2 diabetes (T2D) are uncertain.

**Objective:**

We evaluated the relationship of plasma glucose, GLP-1, and insulin secretion with GE of a 75-g oral glucose load in T2D.

**Design:**

Single-center, cross-sectional, post hoc analysis.

**Setting:**

Institutional research center.

**Participants:**

43 individuals with T2D age 65.6 ± 1.1 years, hemoglobin A1c 7.2 ± 1.0%, median duration of diabetes 5 years managed by diet and/or metformin.

**Intervention:**

Participants consumed the glucose drink radiolabeled with ^99m^Tc-phytate colloid following an overnight fast. GE (scintigraphy), plasma glucose, GLP-1, insulin, and C-peptide were measured between 0 and 180 minutes.

**Main Outcome Measures:**

The relationships of the plasma glucose at 120 minutes, plasma GLP-1, and insulin secretion (calculated by Δinsulin_0-30_/ Δglucose_0-30_ and ΔC-peptide_0-30_/Δglucose_0-30_) with the rate of GE (scintigraphy) were evaluated.

**Results:**

There were positive relationships of plasma glucose at 30 minutes (r = 0.56, *P* < 0.001), 60 minutes (r = 0.57, *P* < 0.001), and 120 minutes (r = 0.51, *P* < 0.001) but not at 180 minutes (r = 0.13, *P* = 0.38), with GE. The 120-minute plasma glucose and GE correlated weakly in multiple regression models adjusting for age, GLP-1, and insulin secretion (*P* = 0.04 and *P* = 0.06, respectively). There was no relationship of plasma GLP-1 with GE. Multiple linear regression analysis indicated that there was no significant effect of GE on insulin secretion.

**Conclusion:**

In T2D, while insulin secretion is the dominant determinant of the 120-minute plasma glucose, GE also correlates. Given the relevance to interpreting the results of an oral glucose tolerance test, this relationship should be evaluated further. There appears to be no direct effect of GE on either GLP-1 or insulin secretion.

Gastric emptying (GE) of solid and liquid nutrients exhibits a wide interindividual variation in health, approximating a linear rate (in the case of solids, following an initial lag phase) ranging between ~1 and 4 kcal/min (1). This variation is even greater in type 2 diabetes (T2D) because of the high prevalence of both accelerated (usually individuals with well-controlled diabetes) (2) and delayed (usually individuals with poorly controlled, complicated, diabetes) GE (3). GE is now appreciated as a major determinant of postprandial glycemia in health, as well as in T2D, accounting for up to 35% of the variance in the initial rise in glucose (4). That certain ethnic groups with a greater propensity for T2D have relatively more rapid GE supports the concept that GE may be a determinant of the risk of diabetes (5).

An understanding of the relationship of glycemia with GE is of potential relevance to the use of the 120-minute blood glucose in an oral glucose tolerance test (OGTT) to diagnose impaired glucose tolerance and T2D (6). The OGTT, despite long-standing concerns about its reproducibility, remains the gold-standard diagnostic technique for this purpose, including for the diagnosis of gestational diabetes (7). Diabetes is classified by OGTT when fasting plasma glucose is ≥7.0 mmol/L or 120-minute plasma glucose is ≥11.1 mmol/L (6). We have reported that there is an inverse relationship between the 120-minute glucose and GE in health and postulated that this reflects a robust, earlier insulin response and intact insulin sensitivity (8, 9). This relationship appears to shift to the right along the spectrum from health to impaired glucose tolerance to T2D, so that the inverse relationship between 120-minute glucose and GE is not evident in individuals with impaired glucose tolerance (8,10). In T2D, we reported that the relationship of glycemia with GE tended to be direct, rather than inverse, but this was not statistically significant, possibly reflecting the modest number (n = 16) of participants studied (8). Given the relevance of this relationship, particularly to the use of the OGTT, the impact of GE on the 120-minute glucose in T2D requires clarification. More recently, it has been suggested that the 60-minute blood glucose in an OGTT may be a better predictor of dysglycemia compared to the 120-minute glucose (11); interestingly, this value is known to be related directly to the rate of GE in individuals with impaired glucose tolerance and T2D but not in healthy individuals (8).

The impact of GE on insulin secretion in T2D is uncertain (12). The latter is known to be influenced by the release of the incretin hormones, glucagon like peptide-1 (GLP-1) and glucose-dependent insulinotropic polypeptide (GIP), following exposure of the small intestine to nutrients (13). In T2D, GLP-1 is of particular relevance given the marked impairment in the insulinotropic capacity of GIP (14). In our studies, which have evaluated the effects of direct intraduodenal infusion of glucose at rates spanning the normal range for GE in health and T2D, GIP secretion was shown to increase in proportion to the rate of nutrient delivery, whereas stimulation of GLP-1 was only evident at a higher threshold (3-4 kcal/min) (15, 16). Information relating to the impact of the rate of GE of glucose on plasma GLP-1 is inconsistent (17); this may reflect the fact that endogenous GLP-1 itself slows GE (18). It has been suggested that in T2D, a sustained increase in beta cell workload from exposure to persistent hyperglycemia may lead to reduced beta cell mass and function (19). Accordingly, in T2D, relatively faster GE, resulting in higher and more sustained postprandial glycemic fluxes, may have the potential to increase beta cell workload and lead to beta cell exhaustion with a consequent reduction in insulin secretory capacity.

Direct measurement of insulin secretion is usually impractical given the requirement for catheterization of the hepatic portal vein (20), and indirect methods use mathematical models to analyze the glucose, C-peptide, and insulin data (21). The ratio of the change in plasma insulin (insulinogenic index) or C-peptide concentration to the change in plasma glucose at 30 minutes during an OGTT is frequently used surrogate markers of insulin secretion (22). In a previous study in health, we reported an inverse relationship of the insulinogenic index with GE (ie, when GE was relatively faster, the insulinogenic index was less) (12).

The primary aims of this study were to address 2 unresolved issues: the relationships of GE of a 75-g oral glucose load with (1) blood glucose at 120 minutes and (2) insulin secretion in T2D.

## Methods

Data were derived from 2 reported studies (23, 24) with respective clinical trial registrations: NCT 02308254 and ACTRN12616001059459.

### Participants

Forty-three Caucasians (14 female, 29 male) with T2D age 65.6 ± 1.1 years, hemoglobin A1c 7.2 ± 1.0%, body mass index 31.6 ± 0.7 kg/m^2^ median duration of known diabetes 5 years, managed by diet and/or metformin only (38 of 43 were taking metformin) were recruited. All participants had T2D, diagnosed by either OGTT and/or measurement of hemoglobin A1c (6). Metformin was withheld for 48 hours prior to each study. None had a history of gastrointestinal disease, previous gastrointestinal surgery, epilepsy, significant alcohol intake (>20 g alcohol/day), or significant cardiac, respiratory, hepatic, and/or renal disease.

### Study Protocol

Participants fasted overnight (14 hours for solids, 12 hours for liquids). On arrival, an intravenous cannula was inserted into an antecubital vein for blood sampling. While seated in front of a gamma camera, participants consumed a 75-g glucose drink (280.5 kcal) dissolved in water (total volume 300 mL) radiolabeled with 20 MBq ^99m^Tc-phytate colloid (Radpharm Scientific, Belconnen, ACT) within 5 minutes. Completion of consumption of the drink was designated as t = 0. Radioisotopic data were acquired for 180 minutes and corrected for γ-ray attenuation, subject movement, and radionuclide decay (25). GE data were acquired in 1-minute frames for the first 60 minutes, followed by 3-minute frames until t = 180 minutes. GE was calculated as the amount of energy emptied (kcal/min) between 0 and 120 minutes. Plasma glucose (measured using the hexokinase technique) (26), total plasma GLP-1 (RRID:AB_2757816, GLPIT-36HK, Millipore, Billerica, MA, USA (27), C-peptide (RRID:AB_2750847, ELISA, 10-1136-01, Mercodia, Uppsala, Sweden) (28), and plasma insulin (RRID:AB_2877672, ELISA, Diagnostics 10-1113, Mercodia, Uppsala, Sweden) (29) were measured at 30, 60, 120, and 180 minutes. Insulin secretion was calculated as Δinsulin_0-30_/Δglucose_0-30_ and ΔC-peptide_0-30_/Δglucose_0-30_ (22). The software used for the statistical analysis was SPSS (IBM) v27, and all analyses were supervised by a professional biostatistician.

### Statistical Analysis

Total areas under the curve (AUCs) between t = 0 and 180 minutes were calculated for GLP-1 and glucose using the trapezoidal rule. Participants were subdivided into 2 groups—those with 120-minute plasma glucose ≥ 11.1 mmol/L and those with a lesser glycemic response—and differences in mean GE rate were compared using Student’s *t*-test.

Multiple linear regression models were used to evaluate the primary outcomes—that is, the relationship of (1) the 120-minute plasma glucose and (2) insulin secretion with GE. In the former, age, GLP-1 AUC, and insulin secretion were the independent variables, and in the latter, age, glucose AUC, and GLP-1 AUC were the independent variables. Direct effects of GE on the 120-minute glucose and insulin secretion were estimated by the standardized partial regression coefficient for GE (β). Scatterplots of all independent variables and covariates against GE indicated that linearity was appropriate for all relationships. Because the residuals from the insulin secretion model showed evidence of nonconstant variance, values were log-transformed for analysis (natural log). Linear regression models were used to evaluate the relationships between glycemic responses (at t = 30, 60, and 180 minutes) and GE.

Each study was independently evaluated for these associations and homogeneity between studies was observed. A *P*-value < 0.05 was considered significant in all analyses. Data are presented as mean ± SE of the mean. The software used for the statistical analysis was SPSS (IBM) v27, and all analyses were supervised by a professional biostatistician.

## Results

**Figure 1.**
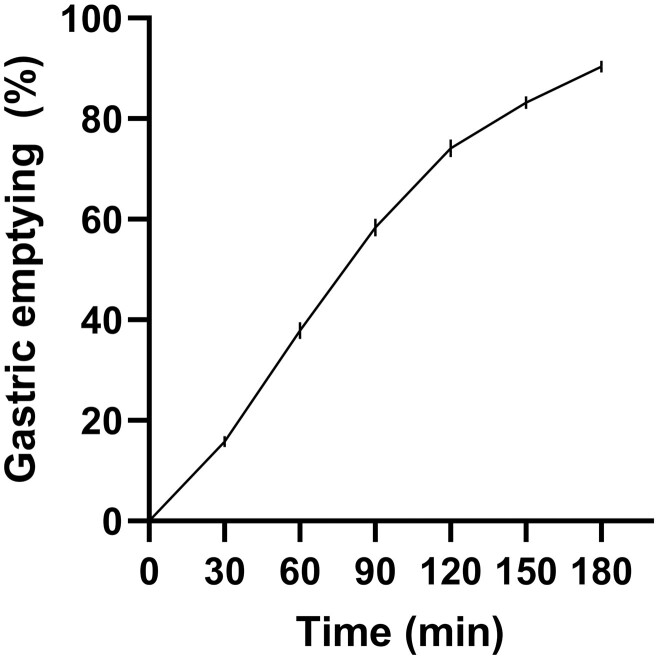
Gastric emptying (expressed as percentage of glucose load emptied) over time in type 2 diabetes (n = 43). Data are mean ± SE of the mean.

All participants tolerated the study well, and in all cases, GE (1.85 ± 0.04 kcal/min) approximated a linear pattern from 0 to 120 minutes. (Fig. 1)

**Figure 2.**
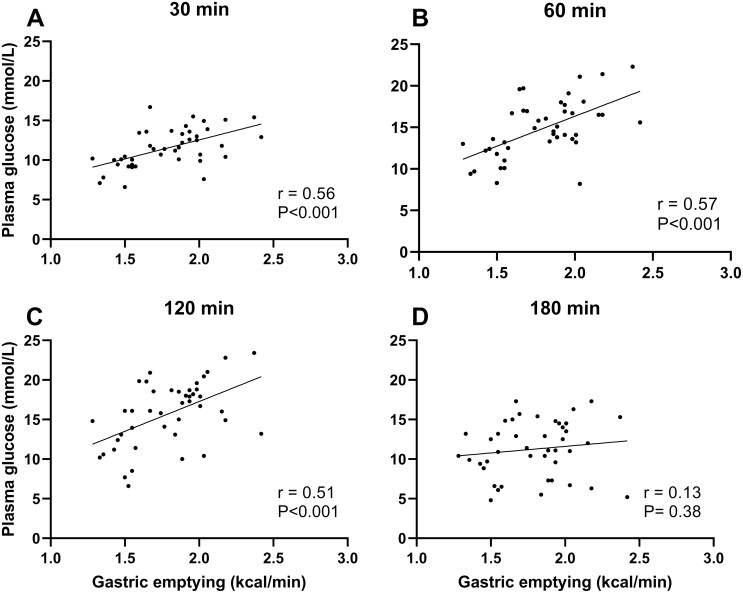
Relationship of plasma glucose at (A) 30, (B) 60, (C) 120, and (D) 180 minutes after a 75-g oral glucose load and gastric emptying (kcal/min) in type 2 diabetes (n = 43).

Fasting glucose was 7.4 ± 0.2 mmol/L, and blood glucose at 120 minutes was 15.7 ± 0.6 mmol/L. Plasma glucose correlated positively and linearly with GE at 30 (r = 0.56, *P* < 0.001), 60 (r = 0.57, *P* < 0.001), and 120 minutes (r = 0.51, *P* < 0.001) but not 180 minutes (r = 0.13, *P* = 0.38). (Fig. 2) In the multiple regression analysis, where age, GLP-1 AUC, and insulin secretion (insulin_0-30_/ Δglucose_0-30_ and C-peptide_0-30_/Δglucose_0-30_) were used as covariates, the 120-minute plasma glucose correlated positively with GE (β = 0.28, *P* = 0.04) when C-peptide_0-30_/ Δglucose_0-30_ was used as the measure of insulin secretion but not significantly with insulin_0-30_/ Δglucose_0-30_ (β = 0.27, *P* = 0.06). (Table 1) Fasting glucose was ≥7.0 mmol/L in 25/43 (58.1%), and at 120 minutes, plasma glucose was ≥ 11.1 mmol/L in 35/43 (81.4%). For the subgroup with the 120-minute plasma glucose ≥ 11.1 mmol/L (n = 35), GE tended to be faster compared to the subgroup with a 120-minute (plasma glucose < 11.1 mmol/L 1.90 ± 0.05 vs 1.65 ± 0.10 kcal/min, *P* = 0.06).

**Table 1.** Multiple regression analysis using the 120-minute blood glucose level as the dependent variable

Model^a^	Unstandardized b	Coefficients SE	Standardized coefficients, beta	Significance	95% CI lower bound	95% CI upper bound
1 (Constant)	2.245	3.588		0.535	−5.001	9.491
Gastric emptying	7.512	1.979	0.510	<0.001	3.515	11.508
						
2 (Constant)	7.777	6.485		0.238	−5.351	20.904
Gastric emptying	3.953	2.063	0.268	0.063	−0.224	8.130
Age, years	0.054	0.076	0.097	0.479	−0.099	0.208
GLP1.AUC180.min	−0.19	0.057	−0.042	0.742	−0.134	0.097
ΔInsulin_0-30_/Δglucose_0-30_	−0.097	0.031	−0.449	0.004	−0.160	−0.034
						
3 (Constant)	9.093	6.401		0.164	−3.864	22.050
Gastric emptying	4.182	1.980	0.284	0.041	0.175	8.190
Age, years	0.048	0.074	0.086	0.521	−0.102	0.198
GLP1.AUC180.min	−0.050	0.056	−0.110	0.381	−0.164	0.064
ΔC-peptide_0-30_/Δglucose_0-30_	−0.022	0.007	−0.471	0.001	−0.036	−0.009

^a^Outcomes of 3 models are provided. In each model, gastric emptying is the first independent variable (Model 1). Model 2 GLP1.AUC180.min: Area under the curve for the concentration of plasma GLP-1 from 0-180 min. includes the other 3 covariates (age, GLP-1, Δinsulin_0-30_/Δglucose_0-30_), and in Model 3, the covariates are age, GLP-1 and ΔC-peptide_0-30_/Δglucose_0-30_.

Baseline fasting GLP-1 was 20.6 ± 1.3 pmol/L, rose to 34.3 ± 2.1 pmol/L at 30 minutes, and then gradually decreased to 31.3 ± 2.0 pmol/L at 60 minutes, 22.9 ± 1.3 pmol/L at 120 minutes, and 16.3 ± 1.0 pmol/L at 180 minutes. There was no significant relationship between GLP-1 and GE at 30 (r = −0.21, *P* = n.s.), 60 (r = −0.09, *P* = n.s.), 120 (r = −0.13, *P* = n.s.), or 180 minutes (r = −0.27, *P* = n.s.).

### Relationship of Insulin Secretion With Gastric Emptying

The analysis of variance test for the model was statistically significant, indicating that there was a relationship between the combinations of GE, age, and GLP-1 AUC with insulin secretion (Δinsulin_0-30_/Δglucose_0-30_) (*P* < 0.001). The addition of the 3 covariates significantly improved the model fit, compared to GE alone (*P* < 0.001). In the final model, the direct effects for GE, age, glucose AUC, and GLP-1 AUC were all negative. The effect for glucose AUC was significant (β = −0.59, *P* < 0.001), while the direct effect of GE on insulin secretion was not (β = −0.13, *P* = 0.39) (Table 2).

**Table 2.** Multiple regression analysis using Δinsulin_0-30_/Δglucose_0-30_ as the dependent variable

Model^a^	Unstandardized b	Coefficients SE	Standardized coefficients, beta	Significance	95% CI lower bound	95% CI upper bound
1 (Constant)	5.231	0.787		<0.001	3.642	6.821
Gastric emptying	−1.357	0.434	−0.439	0.003	−2.234	−0.480
						
2 (Constant)	6.303	1.088		<0.001	4.100	8.506
Gastric emptying	−0.389	0.443	−0.126	0.386	−1.287	0.509
Age, years	−0.007	0.016	−0.064	0.632	−0.039	0.024
Gluc.AUC180.min	−0.164	0.041	−0.588	<0.001	−0.247	−0.081
GLP1.AUC180.min	−0.004	0.012	−0.041	0.746	−0.028	0.020

^a^Model 1 includes gastric emptying as the first independent variable. Model 2 includes the other three covariates (age, glucose, glucagon-like peptide-1). GLP1.AUC180.min: Area under the curve for the concentration of plasma GLP-1 from 0-180 min.

### ΔC-peptide_0-30_/Δglucose_0-30_

The analysis of variance test for the overall model was significant (*P* < 0.001), and the addition of the 3 covariates improved the model fit compared to GE alone (*P* < 0.001). In the final model, the direct effects for GE, age, glucose AUC, and GLP-1 AUC were all negative. The effect for glucose AUC was significant (β = −0.53, *P* = 0.001) while the direct effect of GE on insulin secretion was not (β = −0.07, *P* = 0.63). (Table 3)

**Table 3.** Multiple regression analysis using ΔC-peptide_0-30_/Δglucose_0-30_ as the dependent variable

Model^a^	Unstandardized b	Coefficients SE	Standardized coefficients, beta	Significance	95% CI lower bound	95% CI upper bound
1 (Constant)	322.704	82.056		<0.001	156.987	488.420
Gastric emptying	−111.198	45.262	−0.358	0.018	−202.606	−19.791
						
2 (Constant)	557.810	110.909		<0.001	333.285	782.334
Gastric emptying	−22.019	45.197	−0.071	0.629	−113.516	69.477
Age, years	−2.366	1.583	−0.201	0.143	−5.571	0.839
Gluc.AUC180.min	−14.795	4.192	−0.528	0.001	−23.281	−6.309
GLP1.AUC180.min	−1.564	1.210	−0.163	0.204	−4.014	0.886

^a^Model 1 includes gastric emptying as the first independent variable. Model 2 includes the other three covariates (age, glucose, glucagon-like peptide-1).

## Discussion

We have shown that in T2D, there is a positive relationship between glycemia at 30, 60, and, in particular, at 120 minutes following a 75-g oral glucose load but no significant relationship at any time of the plasma GLP-1 or insulin secretory responses with GE. The correlation between glycemia at 120 minutes with GE is weaker in multiple regression analysis when age, GLP-1, and insulin secretion are covariates.

Consistent with previous reports (2, 8), the plasma glucose concentration following a 75-g oral glucose load was related directly to GE at 30 and 60 minutes in T2D. In the study by Marathe et al (8), there was also a trend for a relationship between glycemia and GE at 120 minutes, but this did not achieve statistical significance likely due to the modest sample size. In health, it has been well established that the relationship of the blood glucose response to a 75-g oral glucose load and GE is direct at 30 minutes, not significant at 60 minutes, and inverse, rather than direct, at 120 minutes (8, 9), presumably reflecting effective glucose counterregulation, particularly because (although it does not appear to be widely appreciated) in the majority of individuals, GE is not completed at that time (ie, an emptying rate ≥ 2.5 kcal/min would be required, which is not usually the case) (30). In contrast, at 180 minutes, GE of a 75-g oral glucose load would be complete in the majority of individuals, which may account for the absence of a relationship with glycemia at that time.

GE is currently not considered in the diagnosis of T2D using the OGTT, including in gestational diabetes where OGTT remains the main diagnostic method (7). Our study indicates that this may represent a limitation; namely, the blood glucose at 120 minutes appears to be dependent on the rate of emptying, which is highly variable between individuals. Moreover, of those for whom the 120-minute blood glucose was not diagnostic of diabetes (18.6%), there was a trend for GE to be slower (*P* = 0.06). This issue, accordingly, warrants further evaluation. It is, however, clear that the timing and direction of the relationship of the glycemic response to oral glucose with GE is dependent on glucose tolerance, demonstrating a shift to the right of the direct relationship with progressive impairment in glucose tolerance (8, 10). The impact of GE on glycemia after a 75-g glucose load is also much more sustained in T2D than in health (ie, there were positive relationships between 30 and 120 minutes whereas in health, the relationships were either nonsignificant or negative after 30 minutes). While this is intuitive and likely to also be the case after carbohydrate-containing meals, further studies are indicated to investigate this. The importance of glucose tolerance is reflected in outcomes of the multiple regression analysis where insulin secretion correlates, not surprisingly, more strongly with the 120-minute plasma glucose than GE, indicating that it is the dominant determinant of the 120-minute plasma glucose, a concept that is widely appreciated. However, our observations also support a lesser appreciated, albeit weaker, relationship of GE with glycemia at 120 minutes. This relationship implies that concomitant use of medications that affect GE, such as opioids, anticholinergics, and GLP-1 receptor agonists may affect the OGTT result.

The absence of a relationship between plasma GLP-1 and GE following oral glucose was clear-cut, consistent with our previous study that found no relationship between GLP-1 and GE following a semisolid meal (31). Intuitively, relatively faster GE may be anticipated to result in more rapid delivery of nutrients to the distal small intestine to stimulate secretion of GLP-1 (4). Moreover, as discussed, our previous studies using direct infusion indicate that GLP-1 secretion in health (16) and T2D (15) is dependent on the rate of intraduodenal glucose delivery. There are several potential explanations for the differences observed. First, in none of the current participants was GE faster than 3 kcal/min, and in our intraduodenal glucose studies, a sustained rise in plasma GLP-1 was only evident when glucose was delivered at >3 kcal/min (15). Second, more rapid GE may not translate to more rapid delivery of glucose to the distal, as opposed to the proximal, small intestine, possibly as a result of the, so-called ileal brake (32) where increased glucose delivery to the distal small intestine slows GE, probably by stimulating GLP-1 and peptide YY (33). Third, studies using the specific GLP-1 antagonist, exendin 9-39, have established that endogenous GLP-1 is a physiological modulator of GE of glucose in health (18, 34); by infusing glucose directly into the small intestine, this mechanism is bypassed.

Our multiple regression analysis indicated that there was no direct relationship between insulin secretion and GE in T2D after adjusting for glucose and GLP-1. We had hypothesized that individuals with relatively faster GE may have a relative reduction in their insulin secretory response if the greater postprandial glycemic excursions leading to beta cell stress, but this was not supported by our findings. A potential explanation is that our cohort comprised individuals with relatively well-controlled T2D, and therefore, insulin secretory function was relatively preserved.

Strengths of our study include the use of scintigraphy, the gold-standard technique to measure GE, and a participant population with similar baseline characteristics. Limitations are that the study population was entirely Caucasian, managed with lifestyle modification and/or metformin monotherapy, and had uncomplicated T2D with reasonable chronic glycemic control as assessed by glycated hemoglobin. Of necessity, we employed surrogate, rather than direct, markers to estimate the insulin secretory response; accordingly, our observations in this area should be viewed circumspectly. While our study failed to show an independent relationship between insulin secretion and GE, the design was cross-sectional, and a prospective study to clarify this issue would be of interest.

## Conclusion

We conclude that in T2D while insulin secretion is a dominant determinant of the glycemic response, relatively faster GE is also associated with an increased glycemic response at 30, 60, and, in particular, 120 minutes following a 75-g oral glucose load, while GLP-1 and insulin secretion do not appear to correlate with GE. These findings should prompt more detailed evaluation of the impact of GE on the diagnostic accuracy of the OGTT. A prospective study to clarify the relevance of GE to the risk of T2D would be of interest.

## Data Availability

Some or all data sets generated during and/or analyzed during the current study are not publicly available but will be provided by the corresponding author on reasonable request.
